# Some southern African plant species used to treat helminth infections in ethnoveterinary medicine have excellent antifungal activities

**DOI:** 10.1186/1472-6882-12-213

**Published:** 2012-11-07

**Authors:** Mathew Adamu, Vinasan Naidoo, Jacobus N Eloff

**Affiliations:** 1 Phytomedicine Programme, Department of Paraclinical Sciences, Faculty of Veterinary Science, University of Pretoria, Private Bag X04, Onderstepoort, Pretoria, 0110, South Africa; 2 Permanent address: Department of Veterinary Parasitology and Entomology College of Veterinary Medicine, University of Agriculture Makurdi, Makurdi, Nigeria

**Keywords:** Antifungal, *Candida*, *Cryptococcus*, *Aspergillus*, Anthelmintic, Therapeutic index, Selectivity, Plant extract, Immune compromised patients

## Abstract

**Background:**

Diseases caused by microorganisms and parasites remain a major challenge globally and particularly in sub-Saharan Africa to man and livestock. Resistance to available antimicrobials and the high cost or unavailability of antimicrobials complicates matters. Many rural people use plants to treat these infections. Because some anthelmintics e.g. benzimidazoles also have good antifungal activity we examined the antifungal activity of extracts of 13 plant species used in southern Africa to treat gastrointestinal helminth infections in livestock and in man.

**Methods:**

Antifungal activity of acetone leaf extracts was determined by serial microdilution with tetrazolium violet as growth indicator against *Aspergillus fumigatus, Cryptococcus neoformans* and *Candida albicans*. These pathogens play an important role in opportunistic infections of immune compromised patients. Cytotoxicity was determined by MTT cellular assay. Therapeutic indices were calculated and selectivity for different pathogens determined. We proposed a method to calculate the relation between microbicidal and microbistatic activities. Total activities for different plant species were calculated.

**Results:**

On the whole, all 13 extracts had good antifungal activities with MIC values as low as 0.02 mg/mL for extracts of *Clausena anisata* against *Aspergillus fumigatus a*nd 0.04 mg/mL for extracts of *Zanthoxylum capense, Clerodendrum glabrum,* and *Milletia grandis*, against *A. fumigatus. Clausena anisata* extracts had the lowest cytotoxicity (LC_50_) of 0.17 mg/mL, a reasonable therapeutic index (2.65) against *A. fumigatus*. It also had selective activity against *A. fumigatus*, an overall fungicidal activity of 98% and a total activity of 3395 mL/g against *A. fumigatus*. This means that 1 g of acetone leaf extract can be diluted to 3.4 litres and it would still inhibit the growth. *Clerodendrum glabrum, Zanthoxylum capense* and *Milletia grandis* extracts also yielded promising results.

**Conclusions:**

Some plant extracts used for treatment of parasitic infections also have good antifungal activity. Because it is much easier to isolate antifungal compounds by bioassay guided fractionation, this approach may facilitate the isolation of anthelmintic compounds from active plant extracts. The viability of this approach can be tested by isolating the antifungal compounds and then determining its anthelmintic activity. Some of these plant extracts may also be useful in combating fungal infections.

## Background

Apart from acting as a source of food, providing shelter and many other uses, plants have been a source of medicine for man and his animals. Infectious diseases caused by bacteria, fungi and viruses continue to be a major health concern especially in sub-Saharan Africa. These organisms cause untold hardship particularly in rural areas due to poor sanitary conditions, limited availability of potable drinking water and poverty. The situation is complicated with the HIV/AIDS pandemic that has ravaged a part of the world with the attendant problem of antimicrobial resistance and opportunistic infections. Even when antimicrobials are available, they remain expensive for the poorer communities and in some cases especially in the rural areas may be adulterated and of little value in the treatment of diseases caused by fungi in man and in animals [1]. Plants have consequently been widely used for the treatment of various ailments in animals. McGaw and Eloff [2] reported that of more than 200 plant species used in ethnoveterinary medicine (EVM) in South Africa, only 27 or 13% have been evaluated for any biological activity. More species need to be evaluated, and more in depth investigation of plants already tested needs to be carried out. Southern African plants are a potential source of undiscovered compounds with high biologically active extracts against a variety of disease-causing organisms [3], or disease-transmitting vectors [4].

The plant species used in this study were chosen based on documented traditional use for anthelmintic purposes. Because some commercial anthelmintics such as benzimidazoles have antifungal activity, we decided to investigate the possible antifungal activity and the cytotoxicity of extracts of these 13 plant species. Acetone was selected as the only solvent based on its ability to extract compounds with a wide range of polarities, its low toxicity in antimicrobial bioassays and because it is easily removed from the extract at low temperature [5,6]. The fungal pathogens were selected based on their importance in opportunistic infections of immune compromised patients.

## Methods

### Plant collection

Leaves of thirteen plant species were collected in November 2009 at the National Botanical Garden in Pretoria, South Africa. The trees were identified and labelled and voucher specimens were kept in the Herbarium of the Garden (Table 1). Plant leaves were dried at room temperature in a well-ventilated room, milled to a fine powder in Macsalab Mill (Model 2000 LAB Eriez®) and stored in closed containers in the dark until use.

**Table 1:** List of plant species used in the investigation, their traditional uses and references

**Plant species**	**Family**	**Medicinal uses**	**Reference**
*Brachylaena discolor*	Asteraceae (267)	Purgatives against intestinal parasites, anthelmintics for calves, sheep and goats	[7-9]
*Zanthoxylum capense*	Rutaceae (96)	Gastric and intestinal disorders, anthelmintics, cough, bronchitis, pleurisy	[7,9]
*Clerodendrum glabrum*	Lamiaceae (403)	Intestinal parasites, coughs, fever and diabetes	[7-9]
*Heteromorpha trifoliata*	Apiaceae (491)	Intestinal worms, colic in horses and vermifuge, enemas for abdominal disorders	[7,8,10]
*Apodytes dimidiata*	Icacinaceae (139)	Enemas for intestinal parasites, purgatives, inflammation of the ear	[7,8,10]
*Strychnos mitis*	Strychnaceae (73)	Malaria, Fevers	[11]
*Maesa lanceolata*	Maesaceae (615)	Anthelmintics, treatment of wounds and infertility	[8]
*Indigofera frutescens*	Papilionaceae (675)	Anthelmintics	[8]
*Leucosidea sericea*	Rosaceae (288)	Treatment of opthalmia, anthelmintics, astringents and vermifuge	[8,12]
*Melia azedarach*	Meliaceae (702)	Effective anthelmintics, emetic, cathartic and treatment of eczema	[8,9,13]
*Clausena anisata*	Rutaceae (317)	Anthelmintics, purgatives, rheumatism, fevers and myiasis	[14]
*Cyathea dregei*	Cyatheaceae (658)	Anthelmintics	[14]
*Milletia grandis*	Papilionaceae (704)	Anthelmintics and tranquilizers	[8,15]

(PRU voucher specimen numbers provided after family names).

### Plant extraction

Plant material (1 g) from each species investigated was extracted with 10 mL of acetone, (technical grade, Merck) in polyester centrifuge tubes [5]. The tube was vigorously shaken for 30 min on an orbital shaker, then centrifuged at 4000 x g for 10 min and the supernatant was filtered using Whatman No.1 filter paper before being transferred into pre-weighed glass containers. This was repeated twice and solvent was removed by evaporation under a stream of air in a fume hood at room temperature to produce the dried extract [16].

### Chromatographic analysis

The extracted chemical components were made up to a concentration of 10 mg/mL and 10 μL was analysed by thin layer chromatography (TLC) separation using aluminium-backed TLC plates (Merck, Silica gel F254). The TLC plates were developed in saturated chambers using mobile phases of varying polarities, namely, ethyl acetate/methanol/water (40:5.4:5) [EMW] (polar/neutral), chloroform/ethyl acetate/formic acid (5:4:1) [CEF] (intermediate polarity/acidic) and benzene/ethanol/ammonia hydroxide (90:10:1) [BEA] (non-polar/basic) [17]. Separated components were examined under UV light at wavelengths of 254 and 365 nm after which TLC plates were sprayed with vanillin-sulphuric acid [18] and heated at 110°C to optimal colour development.

### Antifungal activity

The MIC values were determined using a serial microplate dilution method developed by Eloff [6] and modified by Masoko et al. [19]. Three fungal species, namely *Candida albicans, Cryptococcus neoformans* and *Aspergillus fumigatus* associated with opportunistic infections of immune compromised patients*,* were obtained from the fungal culture collection in the Department of Veterinary Tropical Diseases, Faculty of Veterinary Science at the University of Pretoria. *C. albicans* was isolated from a Goldian finch, *C. neoformans* from a cheetah and *A. fumigatus* from a chicken, all of which suffered from systemic mycosis. None of the animals had been treated prior to sampling. All fungi were maintained in Sabouraud dextrose agar (Oxoid, Basingstoke) until use. For growth inhibition, assays, fungal species were grown overnight in Sabouraud dextrose broth at 35°C prior to the test. Fungi were cultured at 35°C in universal bottles as slants in Sabouraud dextrose agar (Oxoid, Basingstoke) (65 g dissolve in 1 L distilled water and sterilized by autoclaving at 121°C for 30 min). Using sterile cotton swabs the collected conidia were inoculated into Sabouraud dextrose broth (Sigma, Germany) (30 g dissolve in 1 L distilled water and sterilized by autoclaving at 121°C for 30 min) prior to bioactivity assays. Densities of fungal cultures used in bioautography and for MIC determinations were as follows: *C. albicans*, 2.5 × 10^6^ cfu/mL; *C. neoformans*, 2.6 × 10^6^ cfu/mL; *A. fumigatus*, 8.1 × 10^6^ cfu/mL. *Candida albicans* was diluted to a density of about 2.5 × 10^4^ cfu/mL, *C. neoformans*, 2.6 × 10^4^ cfu/mL, and *A. fumigatus* 8.1 × 10^4^ cfu/mL. Tetrazolium violet was used as an indicator of microbial growth [6,19]. Growth of the microorganisms reduces the tetrazolium violet to a red formazan. Amphotericin B was used as a positive control in antifungal activity assays. MIC was registered as the lowest concentration of plant extract inhibiting microbial growth, indicated by a decrease in the intensity of the red colour of the formazan product. The total activity of the extracts was calculated as the total mass (mg) of the extract divided by the MIC value (mg/mL). Total activity value indicates the volume to which the extract derived from 1 g of plant material can be diluted and still inhibits the growth of the microorganism [16,20].

### Cytotoxicity assay using MTT

For this assay Vero monkey kidney cells obtained from a confluent monolayer cells were trypsinised and seeded (0.5 x 10^3^ cells per well) in a 96 well microtitre plate and incubated overnight at 37°C in a 5% CO_2_ atmosphere, in minimal essential medium 200 μl (MEM, Highveld Biological, South Africa) supplemented with 0.1% gentamicin (Virbac^R^) and 5% foetal calf serum (Adcock-Ingram). After 24 hours the media was replaced and 200 μl of the extracts (1, 0.1, 0.01, 0.001 mg/mL) were further incubated for 5 days. Viability of cells was determined using the tetrazolium-based colorimetric MTT assay (3-5-dimethyl thiazol-2-yl-2, 5-diphenyl tetrazolium bromide) described by [21]. In short the media in each well was removed and replaced with fresh media and 30 μl of 5 mg/mL MTT in PBS and subsequently incubated for four hours. Hereafter the medium was removed and cells washed with PBS, prior to the addition of dimethyl sulphoxide (50 μl) to dissolve any formazan crystals present. The absorbance of the wells was measured with a Versamax microplate reader at 570 nm. Different concentrations of berberine chloride (Sigma) were used as a positive control, while wells containing only cells without extracts were the negative control. The percentage cell viability relative to the pure growth was calculated. The LC_50_ values was calculated by determining the concentration of plant extracts resulting in 50% reduction of absorbance compared to untreated cells. Tests were carried out in triplicate and each experiment was repeated three times.

### Bioautographic investigations

For bioautography analysis, thin layer chromatography (TLC) plates were loaded with 10 μL of each extract of 10 mg/mL concentration and dried before developing in mobile phases of BEA, CEF and EMW. The solvent was evaporated from the plates in a stream of air for four days. Plates were then sprayed with concentrated cultures of fungi species in fresh growth medium until completely moist using a spraying gun. The moist plates were incubated at 37°C in an incubator for 24 h. The plates were then sprayed with 2 mg/mL of *p* iodonitrotetrazolium violet (INT) (Sigma) and incubated for a further 12 h. The emergence of purple-red colour resulting from the reduction of INT into its respective formazan was a positive indicator of cell viability. Clear zones against the purple background were indicative of antifungal activity of compounds separated on TLC plates [22].

## Results

### Plant species yield

Different quantities were extracted with acetone from the ground dried leaves. *L. sericea* had the highest percentage yield of 6.27%, followed closely by *A. dimidiata* with 6.07%, and the lowest yield was obtained from *Z. capense* (0.81%). The percentage yields from other dried leaves were: *B. discolor* (3.30), *C. glabrum* (1.60), *H. trifoliata* (1.28), *S. mitis* (3.75), *M. lanceolata* (2.79), *I. frutescens* (2.05), *M. azedarach* (2.29), *C. anisata* (3.40), *C. dregei* (2.50) and *M. grandis* (1.24)*.*

### Phytochemical analysis

**Figure 1:**
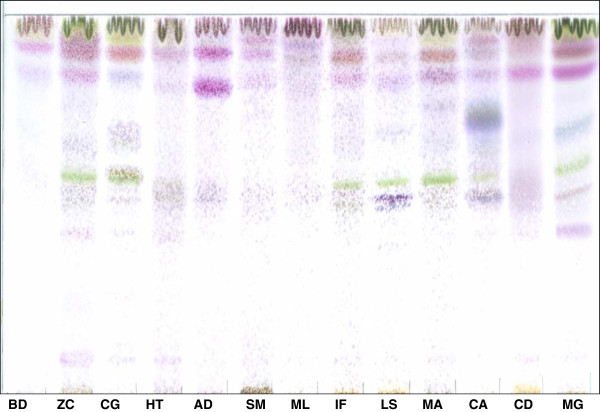
**TLC plates developed in CEF system and sprayed with vanillin sulphuric acid showing varied chemical constituents of the thirteen acetone leaf extracts.** KEY: BD, *B. discolor*; ZC, *Z. capense* ; CG, *C. glabrum*; HT, *H. trifoliata* ; AD, *A. dimidiata* ; SM, *S. mitis* ; ML, *M. lanceolata* ; IF, *I. frutescens* ; LS, *L. sericea* ; MA, *M. azedarach* ; CA, *C. anisata* ; CD, *C. dregei* ; MG, *M. grandis*.

The compounds present in the different extracts that react with the vanillin spray reagent were separated by thin layer chromatography using the CEF solvent system (Figure 1). More than 13 compounds varying in polarity were separated.

### Bioautography

**Figure 2:**
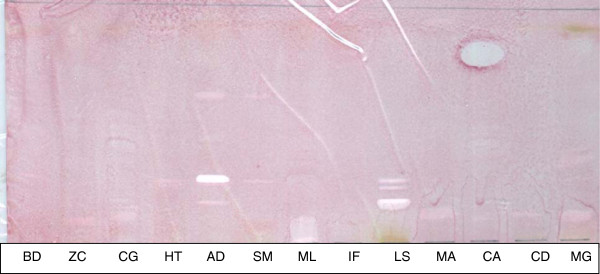
**Bioautogram of thirteen plant leaf acetone extracts separated by the benzene-ethylacetate-ammonia solvent system against *C. albicans *showing antifungal bands.** KEY: BD, *B. discolor*; ZC, *Z. capense* ; CG, *C. glabrum*; HT, *H. trifoliata* ; AD, *A. dimidiata* ; SM, *S. mitis* ; ML, *M. lanceolata* ; IF, *I. frutescens* ; LS, *L. sericea* ; MA, *M. azedarach* ; CA, *C. anisata* ; CD, *C. dregei* ; MG, *M. grandis*.

With the three solvent systems used, 37 active bands were seen for the 3 fungal pathogens in the chromatograms of the different plants extracts. BEA system separated 17 (46%) of the active bands, followed by CEF 14(38%) and EMW 6 (16%) as the least. This indicates that most of the antifungal compounds are non-polar. In the antifungal bioautography, *A. dimidiata* (Rf; 019, 0.37, 0.63) and *L. sericea (*Rf; 0.18, 0.23, 0.28) had 3 active bands each against C. *albicans* in the BEA chromatogram (Figure 2). This was followed by *A. dimidiata* with two active bands in the BEA system against *A. fumigatus.* No antifungal compounds were seen in BEA chromatograms of *B. discolor* and *S. mitis* extracts (Figure 2), CEF and EMW against the fungi organisms used in this study. An active compound with Rf value between 0.93-0.95 was present in *Z. capense* (0.95), *C. glabrum* (0.95)*, M. lanceolata* (0.95)*, I. frutescens* (0.94), *M. azedarach* (0.93) and *C. anisata* (0.93) extracts*.* This compound appears to be a common compound with zone of inhibition shown in all 6 plants listed above. *C. albicans* generally led to the most inhibition bands with the different plant extracts

### Antifungal activity of extracts

MIC values: From the MIC values the average values for the different plant extracts against all three fungi were calculated (Table 2). The average MIC values of all extracts against all three fungi varied between 0.09 and 0.63 mg/mL over the 12 and 24 hour incubation periods. The *Clausena anisata* extract had the best MIC (0.02 mg/mL) against *A. fumigatus.* Extracts of *Z. capense, C. glabrum,* and *M. grandis*, also had excellent antifungal activity (0.04 mg/mL MIC) against *A. fumigatus.* Extracts of *Z. capense, C. glabrum, L. sericea and B. discolor* had good activity (0.08 mg/mL MIC) against *C. neoformans* and also *I. frutescens* extracts against *A. fumigatus* (Table 2)*.*

**Table 2:** **MIC in mg/mL of the leaf extracts of 13 plant species against *A. fumigatus *(AF), *C. albicans *(CA) and *C. neoformans *(CN) incubated for 12h and 24 h**

**Time and fungus**	**12h AF**	**24h AF**	**12h CA**	**24h CA**	**12h CN**	**24h CN**	**Average 12 h**	**Average 24h**	**Degree FCA**
*Brachylaena discolor*	0.31	0.61	0.31	0.31	** 0.08**	0.16	0.23	0.36	65
*Zanthoxylum capense*	** 0.04**	** 0.08**	0.16	0.31	** 0.08**	** 0.08**	** 0.09**	0.16	60
*Clerodendrum glabrum*	** 0.04**	** 0.08**	0.31	0.63	** 0.08**	0.16	0.14	0.29	49
*Heteromorpha trifoliata*	0.63	1.25	0.31	0.63	0.16	0.16	0.37	0.68	54
*Apodytes dimidiata*	0.31	0.31	0.31	0.63	** 0.08**	0.16	0.23	0.37	64
*Strychnos mitis*	0.31	0.63	0.31	0.63	0.63	0.63	0.42	0.63	66
*Maesa lanceolata*	0.31	0.31	0.16	0.31	0.16	0.63	0.21	0.42	50
*Indigofera frutescens*	** 0.08**	0.31	0.16	0.16	0.16	0.16	0.13	0.21	63
*Leucosidea sericea*	0.16	1.25	0.31	0.31	** 0.08**	0.16	0.18	0.57	32
*Melia azedarach*	0.31	0.63	0.16	0.31	0.63	0.63	0.37	0.52	70
*Clausena anisata*	** 0.02**	** 0.04**	0.63	0.63	0.16	0.16	0.27	0.28	98
*Cyathea dregei*	0.16	0.31	1.25	1.25	0.16	0.16	0.52	0.57	91
*Milletia grandis*	** 0.04**	** 0.08**	0.16	0.16	0.63	0.63	0.28	0.29	95
*Average*	0.21	0.45	0.35	0.48	0.24	0.30	0.26	0.41	65.9

Values below 0.1 mg/mL in bold font. Degree of fungicidal activity (FCA) calculated by dividing average of 12 h MIC by average 24 h MIC and multiplying by 100.

Selective activity: If an extract has good activity against several microorganisms it may contain a general metabolic toxin that could also affect animal cells. The *C. anisata* and *M. grandis* extracts had good selective activity against *A. fumigatus*. In other cases some measure of selectivity was present. On average the most sensitive fungus to these extracts was *A. fumigatus* MIC (0.21 and 0.45 mg/mL after 12 and 24 h), followed by *C. neoformans* (0.26 and 0.41 mg/mL) and by *C. albicans* (0.35 and 0.48 mg/mL).

Fungicidal versus fungistatic activity: In an extract with fungistatic activity after a prolonged period growth would again take place. To measure the fungicidal activity we propose that the MIC of the extract after 12 h should be divided by the MIC after 24 h and multiplied by 100 to give the fungicidal activity. The time period would depend on the growth rate of the specific microorganism. The conidia collected and incubated at a high level led to a relatively high growth rate in our case. Extracts of three species *Clausena anisata, Milletia grandis* and *Cyathea dregei* had a fungicidal activity higher than 90% and the *L. sericea* extract had definite fungistatic activity.

The average total activity against the 3 fungal organisms varied from 1026 mL/g for *C. anisata* to 84 mL/g for the *H. trifoliata* extract (Table 3). The highest total activity value of 3395 mL/g against *A. fumigatus* was for the *C. anisata* extract and the lowest was 20 mL/g for *H. trifoliata* against *A. fumigatus* (Table 3). Total activity values indicate which species could be the best source of an extract for use by poor communities or for organic production [20]. *Clausena anisata* with the highest total activity may be a good candidate for further study.

**Table 3:** Total activity of the leaf of 13 plant extract used as anthelmintic screened for antifungal activity using three fungal organisms

	***A. fumigatus***		***C. albicans***		***C. neoformans***	
**Incubation time**	**12**	**24**	**12**	**24**	**12**	**24**
*Brachylaena discolor*	213	108	213	213	825	413
*Zanthoxylum capense*	405	203	101	52	203	203
*Clerodendrum glabrum*	798	399	103	51	199	51
*Heteromorpha trifoliata*	40	20	82	40	159	159
*Apodytes dimidiata*	392	392	392	193	1518	434
*Strychnos mitis*	242	119	242	119	119	144
*Maesa lanceolata*	180	349	349	180	349	89
*Indigofera frutescens*	513	132	256	256	256	256
*Leucosidea sericea*	783	100	404	404	1566	783
*Melia azedarach*	148	73	286	150	73	73
*Clausena anisata*	3395	1698	108	108	424	424
*Cyathea dregei*	313	161	40	40	313	313
*Milletia grandis*	618	309	154	154	39	39

### Cytotoxicity and therapeutic index

*Clausena anisata* had the highest LC_50_ (lowest toxicity) of 0.17 mg/mL, followed by *Melia azedarach* at 0.14 mg/mL. *C. dregei* on the other hand was highly toxic with an LC_50_ of 0.003 mg/mL (Table 4). The ratio between efficacy and safety of an extract is a very important parameter in developing any therapeutic product. The therapeutic index is an indication of the safety of the extract. If there is selective activity to the pathogen the therapeutic index will be higher than 1. Because both values are inversely related to activity the therapeutic index is calculated by dividing the cytotoxicity LC_50_ in mg/mL by the MIC in mg/mL. The higher the value of the therapeutic index the safer the extract. The *Clerodendrum glabrum* extract with a therapeutic index of 4.3 against *A. fumigatus* was the best. The *Clausena anisata* extract was the second best with a therapeutic index of 2.7 against *A. fumigatus* followed by the *Clerodendrum glabrum* extract (2.15) against *C. neoformans*. All other values were below 1 (Table 4).

**Table 4:** **Cytotoxicity (LD**_**50**_**in mg/mL) of extract and selectivity index based on MIC of extract after 12 hours of extracts from thirteen plants examined**

***Plant species***	***Cytotoxicity***	***A. fumigatus***	***C. albicans***	***C. neoformans***
*Brachylaena discolor*	*0.004*	*0.01*	*0.01*	*0.05*
*Zanthoxylum capense*	*0.008*	*0.2*	*0.05*	*0.1*
*Clerodendrum glabrum*	*0.172*	*4.3*	*0.55*	*2.15*
*Heteromorpha trifoliata*	*0.043*	*0.27*	*0.55*	*0.27*
*Apodytes dimidiata*	*0.003*	*0.01*	*0.01*	*0.04*
*Strychnos mitis*	*0.043*	*0.14*	*0.14*	*0.07*
*Maesa lanceolata*	*0.104*	*0.34*	*0.65*	*0.65*
*Indigofera frutescens*	*0.052*	*0.65*	*0.33*	*0.33*
*Leucosidea sericea*	*0.016*	*0.1*	*0.05*	*0.2*
*Melia azedarach*	*0.145*	*0.47*	*0.91*	*0.23*
*Clausena anisata*	*0.053*	*2.65*	*0.08*	*0.33*
*Cyathea dregei*	*0.017*	*0.11*	*0.01*	*0.11*
*Milletia grandis*	*0.021*	*0.53*	*0.13*	*0.03*

Therapeutic index calculated by dividing LD_50_ by MIC.

## Discussion

All 13 plant extracts had some degree of activity against the 3 fungal pathogens used. Many authors consider activity of a plant extract with MIC higher than 0.1 mg/mL as not significant [16,23]. Extracts from 8 of the 13 species with the exception of *H. trifoliata, S. mitis, M. lanceolata, M. azedarach* and *C. dregei* extracts had MIC values lower than 0.1 mg/mL. It may be interesting to correlate the anthelmintic activity of the extracts with their antifungal activity.

*Clausena anisata* extracts had the best activity with an MIC of 0.02 mg/mL. *C. anisata* extracts also had the best antifungal activity when plant extracts of Tanzanian plant species were examined with an MIC value of 1 mg/mL against *C. albicans*[24] compared to 0.63 mg/mL against the same pathogen in the current study. It is satisfying that the results are close despite the use of different extractants, different origins of the plants and different *C. albicans* isolates. The MIC activity of *C. anisata* found here 0.04 mg/mL correlates very well with other published values of 0.05 mg/mL [3]. Bosman et al. [25] reported that *L. sericea* is active against fungi using the non-quantitative disc diffusion method, but no activity was reported for acetone leaf extracts of *L. sericea.* The activity of *L. sericea* dichloromethane, ethanol and petroleum ether extracts against *C. albicans* was very poor with MICs ranging from 1.56-12.5 mg/mL [26] compared to our value of 0.31 mg/mL. The large difference obtained using similar methods may be ascribed to difference in activity of the plants examined.

We have frequently found that acetone is the best extractant for antibacterial and antifungal extracts [5,17,27]. In practically all cases antimicrobial compounds isolated were intermediate polarity compounds. The activity of this plant extract when compared to previous study by Suleiman et al., [3] who reported an MIC value of 0.05 mg/mL against *A. fumigatus* with a total activity value of 2740 mL with the acetone leaf extract of *Loxostylis alata* as against 0.04 mg/mL and 3395 mL total activity in this study remain one of the best activities recorded in our group so far.

The selectivity index of the extracts also helps to select plant extracts that will be useful for further study. In this study, *Clerodendrum glabrum* and *Clausena anisata* with selectivity index of 4.30 and 2.65 respectively, may have the potential for use in *in vivo* animal trials against *A. fumigatus.* The higher the selectivity index the higher the safety of the extract when used *in vivo*. Thus considering the results obtained in this study *C. anisata* and *C. glabrum* should be considered as candidates for further study especially in treatment of poultry infected with *A. fumigatus*.

## Conclusions

After investigating the antifungal activity and cytotoxicity of the different plant extracts, the two most promising plant species for in depth analysis to treat fungal infections were *C. anisata* and *C. glabrum.* The potential use of plant extracts in organic animal production instead of a commercial drug is not a pipe dream. Our group have shown that a *Loxostylus alata* extract was as effective as fluconazole in treating poultry infected with *Aspergillus fumigatus*[28,29]. *C. anisata* and *C. glabrum* extracts had a lower cellular toxicity than the *Loxostylus alata* extract and may deliver an even more useful product that could also be important in treating human patients.

There is a need to develop plant extracts to combat microbial and parasitic infections. Because the bioassays are simple and it is relatively easy to isolate antifungal compounds by bioassay guided fractionation. In this study plants used traditionally to treat helminth infections generally had good antifungal and in some cases excellent antifungal activities. We are in the process of determining the anthelmintic activity of extracts of these traditionally used species. If a good correlation exists between antifungal and anthelmintic activity the search for new anthelmintic compounds or extracts with good anthelmintic activity may be accelerated. The Phytomedicine Programme (http://www.up.ac.za/phyto) has a database in which the antifungal activity of leaf extracts of more than 600 tree species has been determined. If there is indeed a reasonable correlation between antifungal and anthelmintic activities it means that antifungal assays amenable to robotic systems may be used to detect promising candidate plant species to discover anthelmintics.

## Competing interests

The authors declare that they have no competing interests.

## Authors’ contributions

MA participated in the design of the study, carried out field work, prepared the extracts, participated in all assays and wrote the first draft of the manuscript. VN participated in the design and coordination of the study, supervised the study and revised the draft manuscript. JNE conceived the study, participated in the design and coordination of the study, supervised the study, analysed the data and revised the final manuscript. All authors read and approved the final manuscript.

## Pre-publication history

The pre-publication history for this paper can be accessed here:

http://www.biomedcentral.com/1472-6882/12/213/prepub
